# Special Issue “Medical Value of Metal Complexes and Plant-Derived Compounds: Biological Evaluation, Health Effects, Challenges, and Future Opportunities”

**DOI:** 10.3390/ijms26199678

**Published:** 2025-10-04

**Authors:** Agnieszka Ścibior, Manuel Aureliano, Juan Llopis

**Affiliations:** 1 Laboratory of Oxidative Stress, Department of Biomedicine and Environmental Research, The John Paul II Catholic University of Lublin, 20-708 Lublin, Poland; 2 Faculdade de Ciências e Tecnologia (FCT), Campus de Gambelas, Universidade do Algarve, 8005-139 Faro, Portugal; 3 Centro de Ciências do Mar do Algarve (CCMAR/CIMAR LA), Campus de Gambelas, Universidade do Algarve, 8005-139 Faro, Portugal; 4 Department of Physiology, Institute of Nutrition and Food Technology, Biomedical Research Center, Institute of Biosanitary Research, University of Granada, 18071 Granada, Spain; jllopis@ugr.es

## 1. Introduction and Scope

This Special Issue (SI), titled “Medical Value of Metal Complexes and Plant-Derived Compounds: Biological Evaluation, Health Effects, Challenges, and Future Opportunities”, aims to include reports updating our knowledge about the effects of exposure to prevalent heavy metals in the environment, which adversely affect animal and human health. It also focuses on certain natural plant bioactive compounds with multidirectional biological properties, which can effectively alleviate the toxicity of highly hazardous heavy metals and minimize the negative effects of veterinary drugs widely used in the treatment of food-producing animals, on consumer health. It should be emphasized that substances of plant origin are a very interesting research material both in terms of the diversity of the chemical structures of isolated compounds and their possible therapeutic properties [1,2]. For a long time, there has been considerable interest in biologically active compounds of plant origin due to their potential in preventing the toxic effects of xenobiotics [3]. This is a very important and currently relevant trend associated with the search for effective strategies based on natural origin substances aimed at preventing the harmful effects of xenobiotics or mitigating their toxic effects [4].

The biochemical mechanisms of action and the role of plant natural antioxidants remain incompletely understood. For example, quercetin was shown to prevent a specific Ca^2+^-ATPase cysteine oxidation induced by vanadate, but does not reverse ATPase inhibition by the metal species [5]. At the same time, kaempferol administration prevents neurological dysfunctions, most likely through protection against the initial mitochondrial dysfunction [6]. It was further suggested that kaempferol administration can be beneficial not only in preventive treatments, but also in post-insult therapeutic treatments [6].

**Figure 1:**
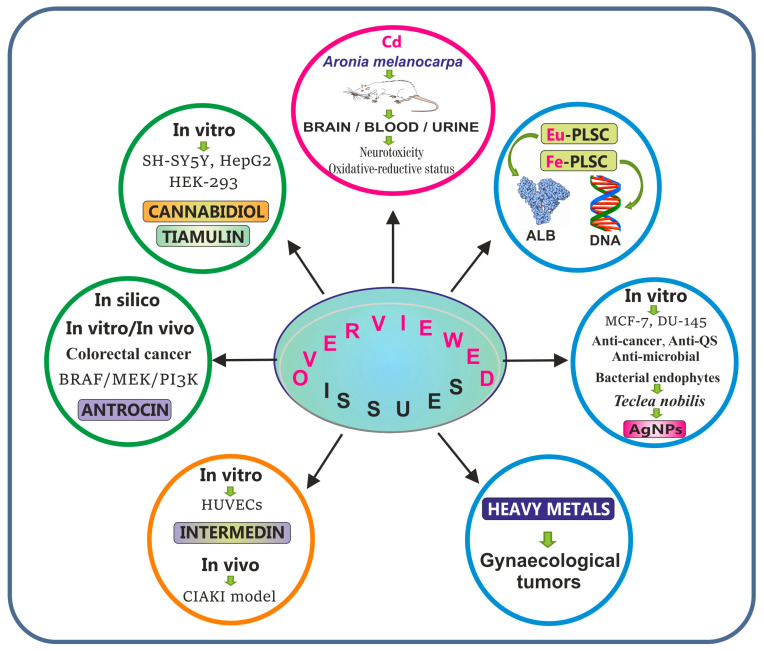
Graphical summary of the included issues: studies on metals and plant-derived compounds (red); studies investigating metals and metal complexes (blue); studies on plant-derived compounds (green); and other studies (orange).

This SI also addresses certain compounds that have the potential as therapeutics for the prevention/treatment of contrast-induced acute kidney injury, as well as for targeting the BRAF/MEK/PI3K oncogenic pathway, which has a critical role in colorectal cancer tumorigenesis, and discusses the role of heavy metals in the pathogenesis of gynecological tumors. Additionally, this SI provides valuable findings on bacterially synthesized silver nanoparticles to demonstrate antibacterial, anti-quorum sensing, and anti-cancer activity, as well as on the structural characterization and biomolecular interaction of europium(III) and iron(III) complexes with the pyridoxal–semicarbazone (PLSC) ligand in terms of future biomedical applications, particularly in drug delivery and DNA targeting. The scientific articles making up this SI, i.e., one review article and six original papers (seven in total), have received over 8000 views (September 2025). Figure 1 summarizes the issues included in this SI.

## 2. Contributions

The contributions were organized into four subsections: studies on metals and plant-derived compounds (one article), studies investigating metals and metal complexes (three articles), studies on plant-derived compounds (two articles), and other studies (one article) (Figure 1).

### 2.1. Metals and Plant-Derived Compounds

The first paper published in this Special Issue, titled “The protective effect of the supplementation with an extract from *Aronia melanocarpa* L. berries against cadmium-induced changes of chosen biomarkers of neurotoxicity in the brain—a study in a rat model of current lifetime human exposure to this toxic heavy metal”, aims to provide thorough knowledge of the damaging impact of cadmium (Cd) on the nervous system in an in vivo model reflecting the current environmental human exposure and information about an effective protective strategy against neurotoxicity of this heavy metal. More precisely, the authors investigated the neurotoxic effect of Cd intake (1 mg and 5 mg Cd/kg of diet) in female Wistar rats for 3, 10, 17, and 24 months and evaluated the action of 0.1% extract of *Aronia melanocarpa* L. against Cd-induced neurotoxicity. They found that exposure to Cd alters the values of various neurotoxicity biomarkers and non-enzymatic proteins crucial for the nervous system’s functioning, as well as the concentrations of some macroelements, such as magnesium and calcium, and some metalloproteinases and their tissue inhibitors in the brain. Simultaneously, they noted that the administration of Cd in conjunction with the extract from *A. melanocarpa* berries alleviated the toxic effects of Cd. The authors concluded that even low-level chronic exposure to Cd may negatively affect the nervous system, and that *A. melanocarpa* berry products can be a potential preventive strategy against Cd-induced neurotoxicity. Additionally, they emphasized the need for further studies on the mechanisms of the protective action of *A. melanocarpa* berries and the need to determine the effective amount that could potentially prevent the population from being exposed to Cd in the future [contribution 1].

The above studies further explored whether the unfavorable effect of Cd and the protective action of *Aronia melanocarpa* L. (Michx.) Elliott berry extract (AME) might be mediated by the impact on the metabolism of bone essential elements such as calcium (Ca) and inorganic phosphorus (P_i_), including pathways regulated by calciotropic hormones [7]. It was suggested that the possible mechanism of the osteoprotective effect of AME during chronic intoxication with Cd involves normalization of the concentrations of calciotropic hormones and improvement in the homeostasis of bone essential elements, providing further evidence that chokeberry products may be an effective strategy in counteracting the unfavorable effects of contaminating metals [7].

### 2.2. Metals and Metal Complexes

One of the original articles included in this SI focuses on anti-microbial properties, anti-quorum sensing, and the anti-cancer potential of silver nanoparticles (AgNPs) synthesized from microorganisms (i.e., *Streptomyces* sp. KE4D and *Bacillus safensis* KE4K) isolated from the Kenyan medicinal plant *Teclea nobilis*. Anti-microbial activity was assessed using agar-well diffusion assays, quorum-sensing inhibition (QSI) was investigated using *Chromobacterium violaceum*, and anti-cancer properties were evaluated against breast (MCF-7) and prostate (DU-145) cancer cell lines using MTT assays. It turned out that KE4D medium 5294 AgNPs showed better QS activity, while KE4K AgNPs demonstrated better anti-microbial properties. Moreover, AgNPs synthesized from *Streptomyces* sp. and *Bacillus safensis* demonstrated dose-dependent cytotoxicity against both cancer cell lines (MCF-7 and DU-145). Based on the obtained results, the authors concluded that AgNPs synthesized from *Streptomyces* sp. and *Bacillus safensis* endophytes show potential in anti-microbial and anti-cancer applications. Simultaneously, they stressed that the bioactivity of biosynthesized AgNPs is influenced by the bacterial isolate and fermentation medium [contribution 2]. From the same research group, previous studies have also been reported describing the antibacterial and anti-QS properties of AgNPs photosynthesized using *Embelia ruminata* leaves, stem bark, and fruit extracts [8].

In turn, an original article written by Jevtovic et al. and included in this SI focused on the structural, computational, and biomolecular interaction of some metal complexes with pyridoxal–semicarbazone (PLSC) ligands, i.e., europium(III) (Eu-PLSC) and iron(III) (Fe-PLSC). The studies conducted by these investigators demonstrated that Eu-PLSC binds more strongly to human serum albumin, while Fe-PLSC has a higher affinity for calf thymus DNA, driven by intercalation. These findings are important because they can contribute to the improvement in future candidates for biomedical applications, especially in the field of drug delivery and DNA targeting [contribution 3]. Recently, this research group has investigated the potential of ultrasound-assisted extracts from Prokupac grape (one of the Balkan Peninsula’s most widely planted red grapes) skins, a wine industry by-product, as functional food ingredients. Four extracts were prepared using different solvents and evaluated for their antioxidant, anti-inflammatory, and anti-microbial properties [9]. It indicated that some extracts exhibited superior antioxidant and anti-inflammatory activities, which was attributed to their high polyphenolic content [9].

Finally, a review on the role of heavy metals in the biology of gynecological neoplasm has been included in the current SI. In this work, the author summarizes the contributions of heavy metals (lead, cadmium, and mercury) and metalloids (arsenic) to the pathogenesis of tumors in the female reproductive system, drawing attention to the gaps in understanding the molecular mechanisms by which these elements contribute to cancer development. Besides the well-known effects of these heavy metals to induce, for instance, DNA damage and enzyme inactivation, thus disrupting critical cellular processes such as growth, proliferation, differentiation, DNA repair, and apoptosis, they mimic endogenous estrogens through interaction with estrogen receptors and cause hormonal disruptions, which is relevant to the pathogenesis of female-related cancers [contribution 4].

### 2.3. Plant-Derived Compounds

Another original article included in this SI focuses on the assessment of the protective action of one of the natural components of hemp (*Cannabis sativa* L.), cannabidiol (CBD). This was investigated as a potential *antidotum* to mitigate the harmful effects of xenobiotics on the toxicity of tiamulin, which is a semi-synthetic antibiotic widely used in veterinary medicine, i.e., in the treatment and prevention of gastrointestinal and respiratory diseases caused by different bacterial pathogens in pigs and poultry (food-producing animals), posing a risk to consumers. The study was conducted in in vitro conditions using three human cell lines as models of neuronal cells (SH-SY5Y), liver cells (HepG2), and kidney cells (HEK-293). CBD was used in two non-toxic concentrations, i.e., 1.56 µg/mL and 3.125 µg/mL. The cytotoxicity of tiamulin and the mixture of CBD and tiamulin were tested after 72 h of incubation. The mitochondrial and lysosomal activity, proliferation, cell membrane integrity, DNA synthesis, oxidative stress, cell death, and cellular morphology were assessed. The nature of interactions between tiamulin and CBD in relation to cytotoxicity against SH-SY5Y, HepG2, and HEK-293 cells was also analyzed. The study showed that long-term exposure of human cells to tiamulin leads to the inhibition of lysosomal (SH-SY5Y) and mitochondrial (HepG2) activity, as well as DNA synthesis (HEK-293). The study also demonstrated that the mixture of CBD with tiamulin reduces oxidative stress, apoptosis, and changes in cell morphology, which provided the basis for the conclusion that the antioxidant and anti-apoptotic activity of CBD may be responsible for the cytoprotective effects of this natural plant substance. Moreover, the interaction between CBD and tiamulin has been noted: an antagonistic (protective) effect at low tiamulin concentrations and a synergistic (increasing the drug’s cytotoxicity) effect at high tiamulin concentrations. The need for further research aimed at recognition of other mechanisms of the protective action of CBD and the need for studies on other cellular models and on animals in order to confirm the in vitro findings was highlighted by the authors [contribution 5]. A recent review from this research group highlighted the association between common *Fusarium* mycotoxins and neurological diseases. It was described that the neurotoxicity mechanisms are mainly related to the increase in oxidative stress in neuronal cells, which leads to higher levels of pro-inflammatory cytokines, such as IL-1β, IL-6, and TNF-α, enzymatic activity, such as GST, GPx, CAT, and SOD, and neurotransmitter dysfunction, such as 5-HT, serotonin, dopamine, and GABA [10]. Several methods to mitigate mycotoxin neurotoxicity using mainly natural substances of plant origin were described [10].

Another study that combined computational and experimental methods and is included in this SI was carried out by Chen and co-workers, who aimed to identify and validate antrocin, a bioactive component isolated from *Antrodia cinnamomea*, as a potential multi-target inhibitor of the BRAF/MEK/PI3K oncogenic pathway in colorectal cancer (CRC). As presented, in silico molecular docking predicted strong binding affinities of antrocin to BRAF, MEK, and PI3K. Furthermore, its drug-likeness and absorption, distribution, metabolism, and excretion properties support antrocin’s potential as a drug candidate. In addition, the in vitro study on two human colorectal cancer cell lines, HCT116 and RKO, validated that the natural sesquiterpene lactone, antrocin, reduced cell viability, spheroid formation, and the migratory ability of both HCT116 and RKO cells, while suppressing the expression of key oncogenic drivers, including BRAF, CD44, MEK, PI3K, KRAS, and AKT in the above-mentioned cells. Moreover, the antrocin-treated tumor-conditioned medium experiments showed that the conditioned medium from antrocin-treated cells was less capable of generating cancer-associated fibroblasts and M2 tumor-associated macrophages. In turn, the preclinical mouse xenograft experiments demonstrated a delay in tumor growth following antrocin treatment. Based on the obtained findings, the authors emphasized that antrocin could be a promising multi-target agent to overcome therapy resistance in CRC [contribution 6].

### 2.4. Other Studies

This SI also includes a study aimed at determining whether intermedin (IMD), a peptide belonging to the calcitonin gene-related peptide family, can mitigate endothelial barrier disruption induced by iodine-based contrast media and exploring the underlying mechanisms involved. The authors used human umbilical vein endothelial cells (HUVECs), which were treated with an iodine-based contrast agent to stimulate kidney injury in vitro, and a rat model of contrast-induced acute kidney injury (CIAKI) to evaluate renal peritubular capillary endothelial cell injury and renal function. Their findings demonstrated that IMD protected against contrast media-induced renal injury in CIAKI. More precisely, this compound reduced peritubular capillary damage and alleviated overall cellular injury from contrast media by activating the cAMP/Rac1 signaling pathway. The authors suggested that IMD has therapeutic potential in CIAKI and emphasized the cAMP/Rac1 pathway as a promising target for preventing CIAKI in at-risk patients [contribution 7].

The authors would like to thank all the contributing authors and reviewers.

## 3. Conclusions and Outlook

The studies described in this SI provide valuable information on the protective action of some natural substances of plant origin against heavy metal- and veterinary drug-induced toxicity and indicate the advisability of conducting further research on the effects of heavy metals and veterinary drugs and their interaction with natural phytochemicals in in vitro and in vivo models. The studies included in this SI also highlight the need for further research on the therapeutic potential of members of the calcitonin family and antrocin, as well as on the role of heavy metals in the pathogenesis of gynecological cancers. Further research is needed on metal–PLSC complexes and the biotherapeutic potential of metal nanoparticles synthesized from endophytic bacteria isolated from medicinal plants.

We believe that the information provided in this SI will stimulate interest among readers in the field of protective strategies against heavy metal- and veterinary drug-induced toxicity. We also believe that the current SI will be valuable to readers interested in bioactive components from *Antrodia cinnamomea* and metal complexes in terms of their biomedical applications, as well as in the contribution of heavy metals to the pathogenesis of gynecological tumors. Furthermore, we think that this SI will be of interest to those interested in compounds that may protect against contrast-induced acute kidney injury, as well as those focused on the potential of endophytic bacteria in the development of innovative therapeutics.

## References

[1] Chaachouay N., Zidane L. (2024). Plant-derived natural products: A source for drug discovery and development. Drugs Drug Candidates.

[2] Gielecińska A., Kciuk M., Mujwar S., Celik I., Kołat D., Kałuzińska-Kołat Ż., Kontek R. (2023). Substances of natural origin in medicine: Plants vs. cancer. Cells.

[3] Thomford N.E., Senthebane D.A., Rowe A., Munro D., Seele P., Maroyi A., Dzobo K. (2018). Natural products for drug discovery in the 21st century: Inovations for novel drug discovery. Int. J. Mol. Sci..

[4] Sobral A.F., Cunha A., Costa I., Silva-Carvalho M., Silva R., Barbosa D.J. (2025). Environmental xenobiotics and epigenetic modifications: Implications for human health and disease. J. Xenobiot..

[5] Fraqueza G., Batista de Carvalho L.A.E., Marques M.P.M., Maia L., Ohlin C.A., Casey W.H., Aureliano M. (2012). Decavanadate, decaniobate, tungstate and molybdate interactions with sarcoplasmic reticulum Ca^2+^-ATPase: Quercetin prevents cysteine oxidation by vanadate but does not reverse ATPase inhibition. Dalton Trans..

[6] Lopez-Sanchez C., Lagoa R., Poejo J., Garcia-Lopez V., Garcia-Martinez V., Gutierrez-Merino C. (2024). An update of kaempferol protection against brain damage induced by ischemia-reperfusion and by 3-nitropropionic acid. Molecules.

[7] Brzóska M.M., Gałażyn-Sidorczuk M., Rogalska J. (2025). The Preventive impact of chokeberry (*Aronia melanocarpa* L.) extract regarding the disruption of calcium and phosphorus homeostasis and chosen pathways of its regulation in an animal model of general population exposure to cadmium. Nutrients.

[8] Rambaran N., Naidoo Y., Mohamed F., Chenia H.Y., Baijnath H. (2024). Antibacterial and anti-quorum sensing properties of silver nanoparticles phytosynthesized using *Embelia ruminata*. Plants.

[9] Avdović E., Dimić D., Nakarada Đ., Simijonović D., Jovičić Milić S., Marković K., Grujović M., Antonijević M., Ćirić A., Milenković D. (2025). Evaluation of bioactive properties of ultrasound-assisted extracts from prokupac grape skins for functional foods. Antioxidants.

[10] Kuć-Szymanek A., Kubik-Machura D., Kościelecka K., Męcik-Kronenberg T., Radko L. (2025). Neurotoxicological effects of some mycotoxins on humans health and methods of neuroprotection. Toxins.

